# Butyrate protects against MRSA pneumonia via regulating gut-lung microbiota and alveolar macrophage M2 polarization

**DOI:** 10.1128/mbio.01987-23

**Published:** 2023-09-27

**Authors:** Yan Zhao, Haoming Sun, Yiwei Chen, Qiang Niu, Yiting Dong, Mei Li, Ye Yuan, Xiaojun Yang, Qingzhu Sun

**Affiliations:** 1 College of Animal Science and Technology, Northwest A&F University, Yangling, Shaanxi, China; Harvard Medical School, Boston, Massachusetts, USA

**Keywords:** MRSA pneumonia, lung microbiota, gut microbiota, sodium butyrate, alveolar macrophage, M2 polarization

## Abstract

Methicillin-resistant *Staphylococcus aureus* (MRSA) is a well-recognized cause of bacterial pneumonia in general. The gut microbiota and their metabolic byproducts act as important modulators of the gut-lung axis. Our investigation indicates a significant reduction in the abundance of butyrate producer unclassified_f__*Lachnospiraceae* within the lung and gut microbiota of MRSA-infected mice, as well as a significant decrease in the levels of butyrate in gut and serum. Additionally, supplementary sodium butyrate (NaB) significantly reduces bacteria colonization in the lung, suppresses pro-inflammatory cytokines expression, and enhances lung tissue morphology in MRSA-treated mice. The results of high-throughput 16S rDNA sequencing demonstrate that NaB reshapes the gut and lung microbiota by drastically reducing the abundance of potential pathogenic bacteria in the gut and cell motility-related bacteria in the lung, which are induced by MRSA. Moreover, NaB treatment augments the gut and circulating butyrate levels. Mechanistically, NaB promotes signal transducer and activator of transcription 1 (STAT1) acetylation and inhibits dimer STAT1 phosphorylation by reducing the binding of histone deacetylase 3 to STAT1, thereby altering alveolar macrophage polarization toward the M2 phenotype. Collectively, our findings suggest that NaB exerts a preventative effect against MRSA-induced pneumonia by enhancing the gut-lung microbiota and promoting macrophage polarization toward an anti-inflammatory M2 phenotype. The prophylactic administration of NaB emerges as a promising strategy for combating MRSA pneumonia.

**IMPORTANCE:**

Pneumonia caused by methicillin-resistant *Staphylococcus aureus* (MRSA) continues to carry a high burden in terms of mortality. With the roles of gut microbiota in mediating lung diseases being gradually uncovered, the details of the molecular mechanism of the “gut-lung axis” mediated by beneficial microorganisms and small-molecule metabolites have gradually attracted the attention of researchers. However, further studies are still necessary to determine the efficacy of microbial-based interventions. Our findings indicate that sodium butyrate (NaB) alleviates MRSA-induced pulmonary inflammation by improving gut-lung microbiota and promoting M2 polarization of alveolar macrophages. Therefore, the preventive administration of NaB might be explored as an effective strategy to control MRSA pneumonia.

## INTRODUCTION

Pneumonia remains a major infectious cause of hospital-acquired infection and mortality worldwide (1, 2). *Staphylococcus aureus* is one of the most notorious and widespread bacterial pathogens, and also a serious cause of pneumonia (3). The situation is further compounded by the emergence of methicillin-resistant *S. aureus* (MRSA), responsible for 10%–40% of pneumonia cases and associated with substantial morbidity and mortality (4, 5). Given the escalating prevalence of antibiotic-resistant bacteria, the effective management of MRSA pneumonia has become a pressing global concern, necessitating the development of novel preventative and therapeutic strategies against MRSA infection.

The intestinal microbiota plays a fundamental role in shaping human health and is a critical factor in modulating gut physiology and extraintestinal functions (6). Extensive research has demonstrated the potential of targeting the gut microbiota in therapeutic approaches for the prevention and treatment of respiratory diseases. Metabolites of gut microbes, such as short-chain fatty acids (SCFAs), have been identified as pivotal mediators of the gut-lung interplay (7). Among these metabolites, butyric acid and its derivatives are widely used in chemical industry, food, feed, medicine, and other fields (8 – 10). Studies on mice models indicate that butyrate administration can provide energy for colonic epithelial cells, thereby contributing to intestinal barrier integrity, regulating energy absorption and immune response (11 – 13). Therefore, the administration of butyrate represents an attractive strategy for the microbiota-targeted intervention to achieve targeted health outcomes. About 2% of luminal butyrate enters the circulation through the portal vein, subsequently influencing immune responses in distance tissues (14). Previous studies have indicated that sodium butyrate (NaB) inhibits the inflammation of lipopolysaccharide (LPS)-induced acute lung injury (ALI) by regulating the toll-like receptors 4 (TLR4)/nuclear factor kappa-B (NF-κB) signaling pathway (15). According to Li et al., NaB alleviates LPS-induced acute lung injury in mice via inhibiting high mobility group protein B1 release (16). However, limited information exists regarding the anti-inflammatory mechanisms of butyrate in pneumonia induced by gram-positive bacteria.

The lung is characterized by a specialized milieu with individual microbial flora (17 – 19). The lung microbiota undergoes alteration in numerous respiratory disorders such as obstructive airway diseases, interstitial lung diseases, infections, and lung cancer (20 – 22). The bi-directional gut-lung axis connecting the intestinal and pulmonary microbiota is widely accepted, and diet could play a crucial role in modulating both microbiota in healthy and pathological states (23). Nutrition interventions and the administration of probiotics or prebiotics can be therapeutically targeted to improve the outcomes of respiratory disorders. A fiber-rich diet simultaneously influences the intestinal and lung microbiota, indicating diet influence on lung immunity (24). Fiber intake increases SCFA levels in blood and has been reported to provide protection against allergic inflammation in lung and reduce mortality related to respiratory diseases (25). However, the full understanding of how SCFAs, specifically butyrate, orchestrate immune cell behavior and influence the development of lung diseases remains obscure.

Alveolar macrophages (AMs) play a critical role in regulating the immune response to pathogens and maintaining tissue homeostasis (26). The plasticity of macrophages enables them to generate tailored responses to local microenvironmental stimuli (27). However, the function and modulation of the gut-lung axis on AMs, particularly on macrophage polarization in MRSA pneumonia, remain incompletely elucidated. Thus, we sought to analyze the impact of butyrate administration on MRSA pneumonia and macrophage polarization. We also aimed to investigate the role of the lung and gut microbiota in mediating the effects of butyrate on host lung inflammation.

## RESULTS

### Pulmonary exposure to MRSA results in perturbations of the gut-lung microbiota and a reduction of butyrate levels

**Fig 1:**
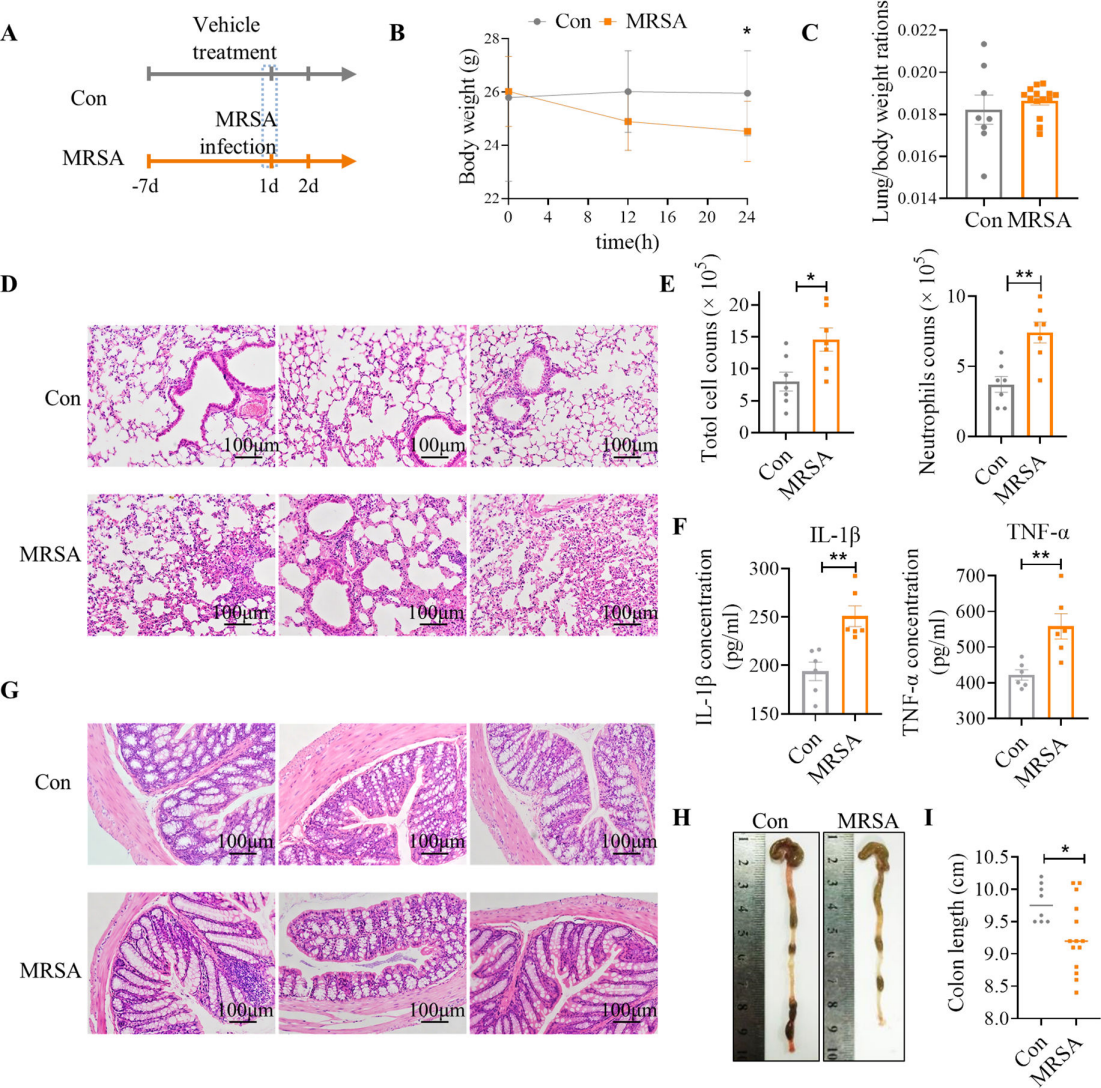
The establishment of MRSA pneumonia. (**A**) Schematic diagram of the experimental design. (**B**) Body weight changes of mice within 24 h of Con (*n* = 8) and MRSA groups (*n* = 14). (**C**) The lung to body weight ratio. (**D**) Representative HE staining section of lung from three mice in each group. (**E**) The total cells and neutrophils in BALF were measured by Wright-Giemsa staining (*n* = 7). (**F**) ELISA results for IL-1β and TNF-α cytokine expression in lung (*n* = 6). (**G**) Representative HE staining section of colon from three mice in each group. (**H**) Representative macroscopic observations of colon length. (**I**) Quantitative reduction of colon length (*n* = 8–14). *, *P* < 0.05; **, *P*  <  0.01, were calculated by Student’s *t*-test.

To first gain insight into the influence of lung microbiota and potential response of intestinal microbiota during MRSA pneumonia, mice were intratracheally challenged with MRSA (5 × 10^6^ colony forming units (CFU) (Fig. 1A). The weight of mice significantly decreased within 24 h after MRSA infection, but the lung body ratio did not change significantly (Fig. 1B and C). In comparison to the control (Con) group, mice treated with MRSA displayed inflammatory cell infiltration in the lungs (Fig. 1D), along with a significant increase in total cell and neutrophil counts in the bronchoalveolar lavage fluid (BALF) (Fig. 1E). MRSA infection resulted in a significant increase in the concentration of the inflammatory cytokines tumor necrosis factor α (TNF-α) and interleukin-1β (IL-1β) in lungs (Fig. 1F). Importantly, it was observed that the colon length of mice infected with MRSA was significantly shorter than that of the control mice (Fig. 1H and I). Histopathological examination of the colon using hematoxylin and eosin (HE) staining revealed inflammatory cell infiltration, reduced goblet cells, thinning of the muscle layer, and varying degrees of expansion in MRSA-infected mice (Fig. 1G). The enzyme linked immunosorbent assay (ELISA) results revealed elevated levels of the inflammatory cytokine IL-1β in both the serum and colon of mice with MRSA lung infection, though the observed increase did not reach statistical significance (Fig. S1A and B). In addition, the use of selective culture medium for *S. aureus* plate counting indicated that *S. aureus* did not migrate to the bloodstream following lung infection (Fig. S1C).

**Fig 2:**
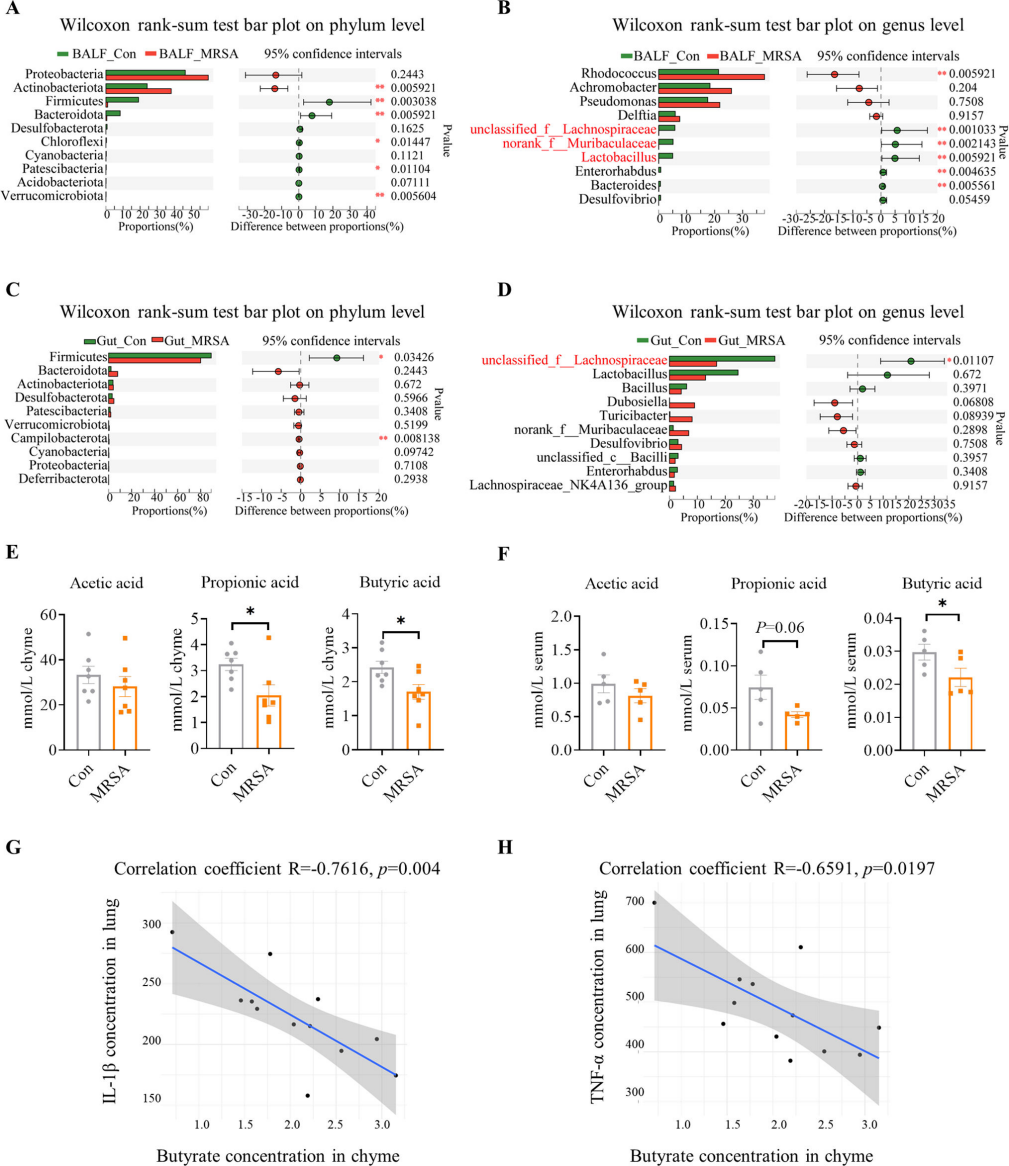
Changes in the lung and gut microbiota as well as SCFAs in mice with MRSA-induced pneumonia. (**A–B**) Relative abundance comparison of top 10 phyla and genera in the lung microbiota. (**C–D**) Relative abundance comparison of top 10 phyla and genera in the gut microbiota. (**E–F**) The concentrations of SCFAs in chyme and serum. (**G–H**) Spearman correlation analysis between the concentration of inflammation cytokines (IL-1β, TNF-α) in the lung and the concentration of butyrate in the chyme. *R* represents the coefficient correlation. * 0.01 < *P* ≤ 0.05, ** 0.001 < *P* ≤ 0.01.

To further explore the alteration of lung microbiota and the response of gut microbiota to bacterial pneumonia, the diversity and composition of BALF and gut microbiota were tested by 16S rDNA sequencing. The α-diversity indexes of the lung microbiota, including Chao and Shannon, were markedly reduced in MRSA-infected mice (Fig. S2A). The core microbiota at the phylum and genus levels was identified to estimate the dysfunctional bacterial groups associated with pneumonia (Fig. S3A). In contrast to the Con group, mice with MRSA pneumonia demonstrated markedly lower levels of the prominent phyla *Firmicutes* and *Bacteroidota* in the lung microbiota, which are typically abundant in the lung microbiota of healthy individuals (Fig. 2A). Furthermore, MRSA-infected mice exhibited reduced relative abundances of several protective bacterial genera, including unclassified_f__*Lachnospiraceae*, *Lactobacillus*, and norank_f__*Muribaculaceae* in lungs compared to healthy controls (Fig. 2B).

Unlike the lung microbiota, the gut microbiota exhibited a significant increase in α-diversity (Shannon index) during MRSA-induced pneumonia (Fig. S2B). Additionally, a distinct separation in β-diversity was observed between the two groups (Fig. S2C). The dominant phyla in the gut microbiota were *Firmicutes* and *Bacteroides*, collectively accounting for over 90% of all phyla (Fig. S3B). Mice infected with MRSA pneumonia demonstrated a reduced abundance of *Firmicutes* in the gut when compared to healthy controls (Fig. 2C). The relative abundance of unclassified_f__*Lachnospiraceae* decreased in both the lungs (Fig. 2B) and the guts (Fig. 2D), accounting for 5.71% and 38.11% of the whole microbiota, respectively. unclassified_f__*Lachnospiraceae* belong to *Lachnospiraceae* which are involved in metabolism as butyrate producer (28). Moreover, a noteworthy reduction in butyric acid levels was observed in the serum and chyme of mice with MRSA pneumonia (Fig. 2E and F). Spearman correlation analysis revealed a negative correlation between the concentration of butyric acid in chyme and the concentration of IL-1β and TNF-α in the lungs (Fig. 2G and H). These findings indicate that MRSA pneumonia disrupts the gut-lung microbiota, leading to a significant decrease in the abundance of butyric acid-producing microorganisms.

### Sodium butyrate pretreatment prevents mice from MRSA-induced pneumonia

**Fig 3:**
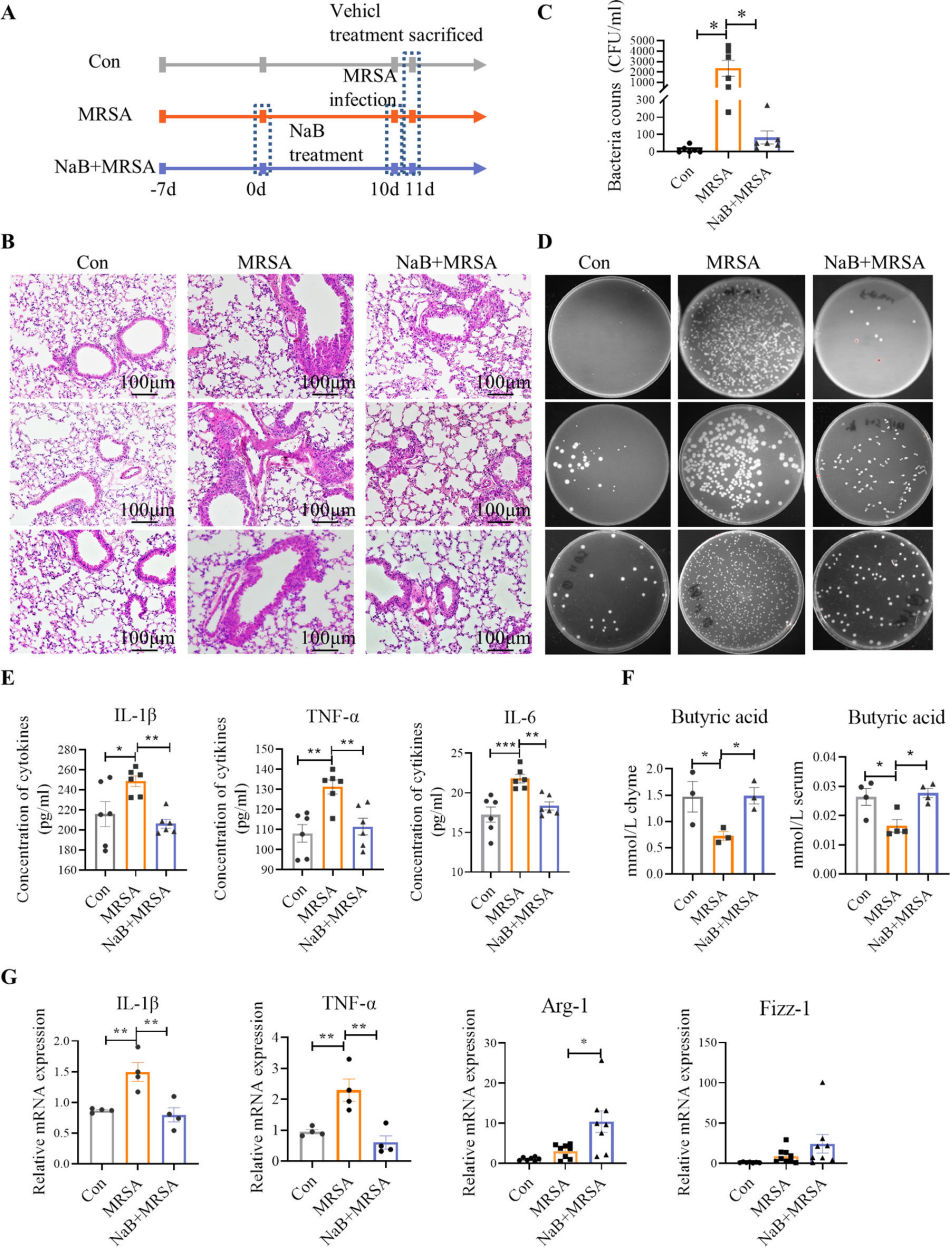
Sodium butyrate pretreatment prevents mice from MRSA-induced pneumonia. (**A**) Schematic diagram of the experimental design. (**B**) Representative HE staining section of lung from three mice in each group. (**D**) The representative colonies of bacteria formed on tryptic soy broth (TSB) agar plates in BALF from three mice in each group (**C**) is the quantitative result. (**E**) ELISA results for IL-1β, TNF-α, and IL-6 cytokine expression in lung (*n* = 6). (**F**) The chime and serum levels of butyric acid in mice were measured by gas chromatography (*n* = 4). (**G**) Transcriptional levels of macrophage polarization markers, including IL-1β, TNF-α, Arg-1, and Fizz-1 were assessed in lung tissue (*n* = 4–8). *, *P* < 0.05, **, *P*  <  0.01, and **, and *P*  <  0.01 were calculated by one-way analysis of variance (ANOVA) with Duncan’s post hoc test.

We further investigated the impact of intestinal intervention with NaB on pneumonia. Mice were treated with NaB orally via gavage for 10 days before MRSA exposure (Fig. 3A). HE staining demonstrated that MRSA-infected lungs displayed inflammatory cell infiltration, airway wall cell hyperplasia, and alveolar thickening, which were alleviated by NaB pretreatment (Fig. 3B). Furthermore, mice subjected to NaB pretreatment displayed a significant reduction in bacterial CFU (Fig. 3C and D) and a decrease in the concentration of inflammatory cytokines (IL-1β, TNF-α, and IL-6) in comparison to MRSA-infected mice (Fig. 3E). Pretreatment with sodium butyrate significantly increased the levels of butyrate in the gut and serum (Fig. 3F). Additionally, compared with the group infected with MRSA alone, NaB pretreatment resulted in a noteworthy downregulation of the transcriptional levels of M1 macrophage factors (IL-1β, TNF-α), while concurrently upregulating the level of the M2 macrophage factor arginase-1 (Arg-1) in lung tissue (Fig. 3G).

### Sodium butyrate protected against MRSA pneumonia through gut-lung microbiota

**Fig 4:**
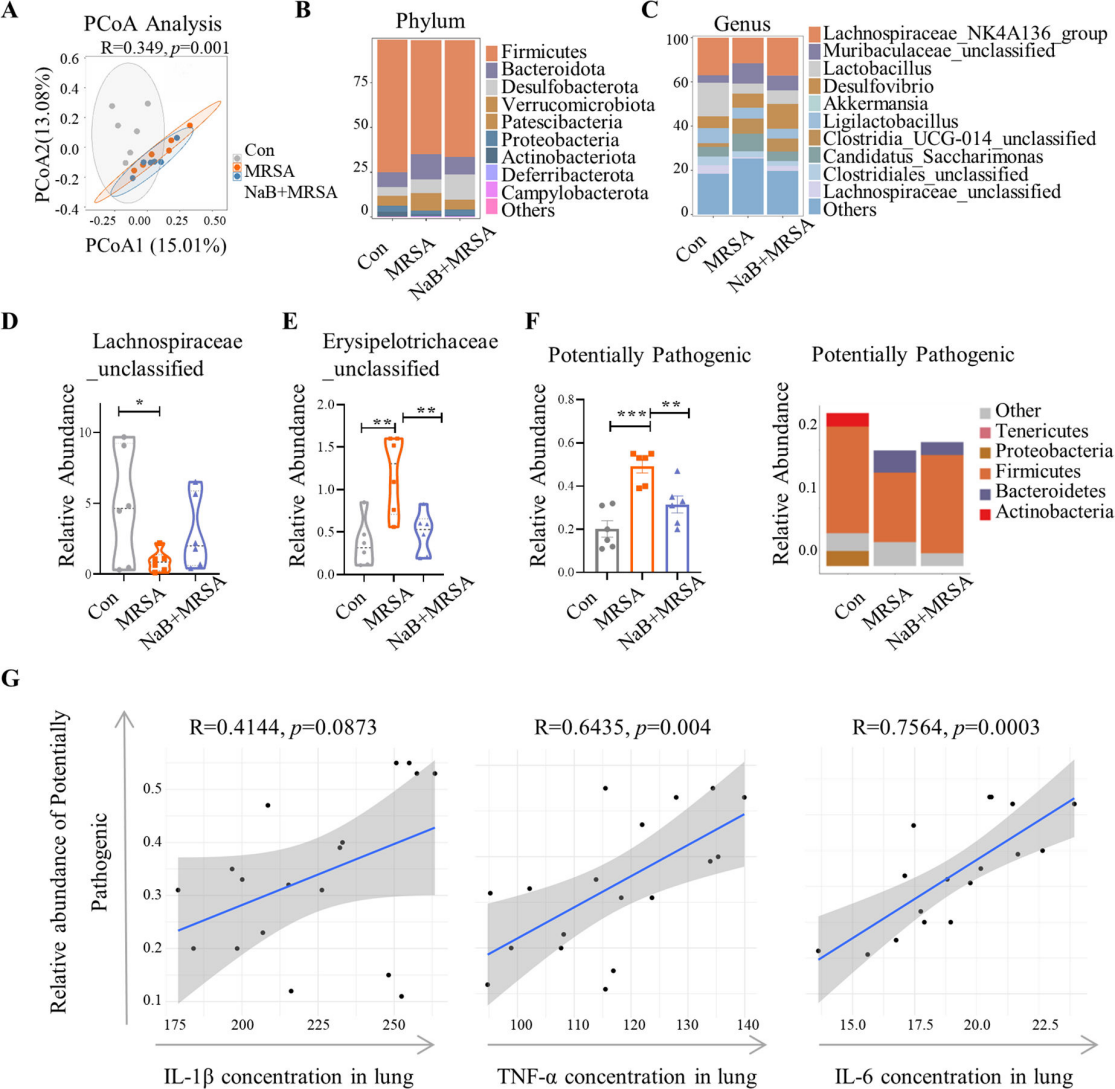
Effects NaB pretreatment on gut microbiota. Cecum chyme of mice in three groups was collected for 16S rDNA sequencing. (**A**) Principal co-ordinates analysis (PCoA) based on the unweighted UniFrac analysis of amplicon sequence variant (ASV). (**B–C**) The top 15 bacteria with maximum abundance of gut microbiota at the phylum and genus levels. (**D–E**) Differences among groups were found in the relative abundance of *Lachnospiraceae*_unclassified and *Erysipelotrichaceae*_unclassified. (**F**) The histogram showed the phenotypes as potentially pathogenic from each group based on BugBase. The stacked bar shows the relative abundance of top 5 phyla in different phenotypes of each group. (**G**) Spearman correlation analysis between the concentration of inflammation cytokines (IL-1β, TNF-α, IL-6) in the lung and relative abundance of potentially pathogenic in the gut. *n* = 6/diet group. *, *P* < 0.05, **, *P* < 0.01, and ***, *P* < 0.001 were calculated by one-way ANOVA with Duncan’s post hoc test.

Previous studies have demonstrated that NaB restored the imbalanced gut microbiota induced by a high-fat diet in mice (29). To investigate the impact of NaB on the microbiota of mice with MRSA-induced pneumonia, we conducted 16S rDNA sequencing on cecum chyme and BALF. Consistent with the findings observed in the MRSA pneumonia model (Fig. S2C), the β-diversity analysis of the gut microbiota revealed a marked separation between the Con group and the MRSA group (Fig. 4A). The specific changes of bacteria at different taxonomic levels were further compared among the three groups (Fig. 4B and C). Though pretreatment with NaB failed to mitigate this alteration (Fig. 4A), MRSA infection resulted in decreased levels of *Firmicutes* and *Lachnospiraceae*_unclassified, as well as an elevated level of *Erysipelotrichaceae*_unclassified in the gut. However, NaB pretreatment attenuated the alteration in the abundance of *Erysipelotrichaceae*_unclassified (Fig. 4D and E). The microbiome phenotypes, as predicted by BugBase database (30), showed a significantly higher abundance of predicted pathogenic potential microbes in the gut of MRSA-treated mice, but NaB pretreatment reversed this alteration (Fig. 4F). Furthermore, a statistically significant positive correlation was observed between the abundance of potential pathogenic bacteria in the gut and the concentrations of inflammatory cytokines in the lungs across all three groups (Fig. 4G). These findings suggest that NaB possesses the capacity to alleviate lung inflammation by regulating the abundance of potentially pathogenic bacteria in the gut.

The BALF samples were collected to verify the lung microbial profile. The total feature number of lung microbiota in MRSA group decreased significantly, which increased in NaB group slightly, but not significantly (Fig. S4A). Both α-diversity (Chao1 index) and β-diversity among the Con, MARS, and NaB + MARS mice showed distinct changes (Fig. S4B and C). To explore the overall variation in lung microbiota composition among three groups, the community difference at phylum and genus level was compared (Fig. S4D and E). Prophylactic intake of NaB can reverse the dysbiosis at the phylum level in the lung microbiota induced by MRSA (Fig. S4D). The level 2 Kyoto Encyclopedia of Genes and Genomes (KEGG) pathways indicated a low abundance of bacteria related to cell motility in MRSA group, which was restored to the normal levels in NaB pretreatment group (Fig. S4F). These findings suggest that NaB mitigates lung inflammation by modulating the gut-lung microbiota.

### Sodium butyrate inhibits the phosphorylation of STAT1 in alveolar macrophages stimulated by MRSA culture supernatant

**Fig 5:**
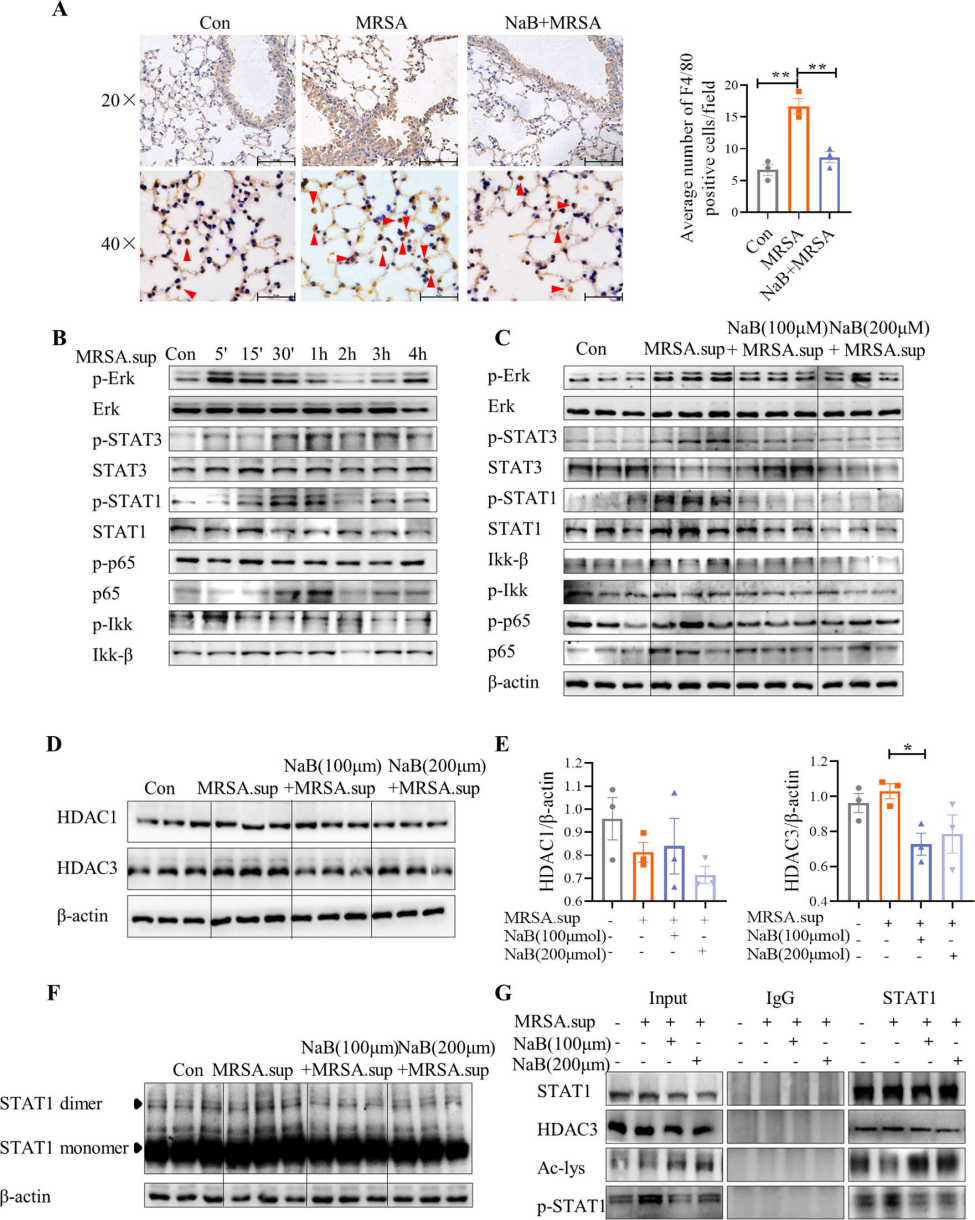
NaB suppresses MRSA.sup-induced MH-S phosphorylation of the STAT1 through histone deacetylase 3 (HDAC3). (**A**) Representative image of immunohistochemistry staining for F4/80 + macrophage in the lung from Con, MRSA, and NaB + MRSA groups, and graphs of F4/80 + immunostaining scores. MH-S cells, the mouse lung alveolar macrophages, were treated with MRSA medium supernatant (MRSA.sup) for 30 min. (**B**) Representative western blot bands of time course of inflammation related proteins in MRSA.sup-induced MH-S. Cells were pretreated with NaB (100 µmol, 200 µmol) for 3  h prior to treatment of MRSA.sup for 30  min. (**C**) Representative western blot bands of inflammation related proteins in MRSA.sup- and NaB + MRSA.sup-induced MH-S. (**D–E**) Representative western blot images and quantitative analyses of the HDAC1 and HDAC3 after treatment with either MRSA.sup or NaB + MRSA.sup. (**F**) Western blot of the dimer conformations of STAT1 in MRSA.sup or NaB + MRSA.sup group. (**G**) Cells were pretreated with NaB for 3  h prior to treatment of MRSA.sup for 30  min, and subjected to co-immunoprecipitation (Co-IP) with an anti-STAT1 antibody, followed by western blot analysis using antibodies against STAT1, P-STAT1, acetyl lysine (Ac-lys) or HDAC3, respectively. *, *P* < 0.05 and **, *P* < 0.01 were calculated by one-way ANOVA with Duncan’s post hoc test.

Anti-F4/80 staining of histological sections confirmed that the lung tissue was infiltrated by AMs following intratracheal injection of MRSA, which was mitigated by NaB pretreatment (Fig. 5A). Subsequently, we examined the activation of the inflammation-related signaling pathway in the mouse alveolar macrophage line MH-S by supernatant of MRSA (MRSA.sup) medium or heat-inactivated MRSA at different time points (Fig. 5B; Fig. S5A). The results indicated that MRSA.sup activated phosphorylation of STAT3 and STAT1 at 30 min, as well as extracellular regulated protein kinases (ERK) phosphorylation at 5 min in MH-S cells. NaB induced the expression of inflammation-related proteins at high concentrations (500 µmol, 1,000 µmol, 5,000 µmol), but not at low concentrations (50 µmol, 100 µmol, 200 µmol) (Fig. S5B). NaB (100 µmol, 200 µmol) pretreatment did not affect the expressions of NF-κB signaling pathway, but significantly inhibited the phosphorylation of STAT1 in MH-S cells (Fig. 5C; Fig. S5C).

The primary mechanisms of NaB action involve binding to G protein-coupled receptors (GPCRs) or inhibiting histone deacetylases (HDACs). Pretreatment with NaB did not alter the three major GPCRs (Fig. S5D), leading us to suspect that NaB acts via HDACs. NaB pretreatment markedly reduced histone deacetylase 3 (HDAC3) expression, but not HDAC1 (Fig. 5D and E). Previous research has shown that HDAC3 strongly counteracts histone acetylase CREB (cyclic adenosine monophosphate response element binding protein) binding protein (CBP)-mediated STAT1 acetylation, thereby activating STAT1 signaling (31). Our results demonstrated that NaB pretreatment significantly reduced the formation of STAT1 dimers in MH-S cells (Fig. 5F). Co-immunoprecipitation (Co-IP) results indicated that NaB pretreatment considerably diminished the binding of STAT1 and HDAC3, enhanced STAT1 acetylation, and reduced STAT1 phosphorylation (Fig. 5G), strongly indicating that NaB diminishes STAT1 phosphorylation via promoting HDAC3-catalyzed STAT1 acetylation.

### Sodium butyrate promotes macrophage polarization toward M2 phenotype by suppressing STAT1

**Fig 6:**
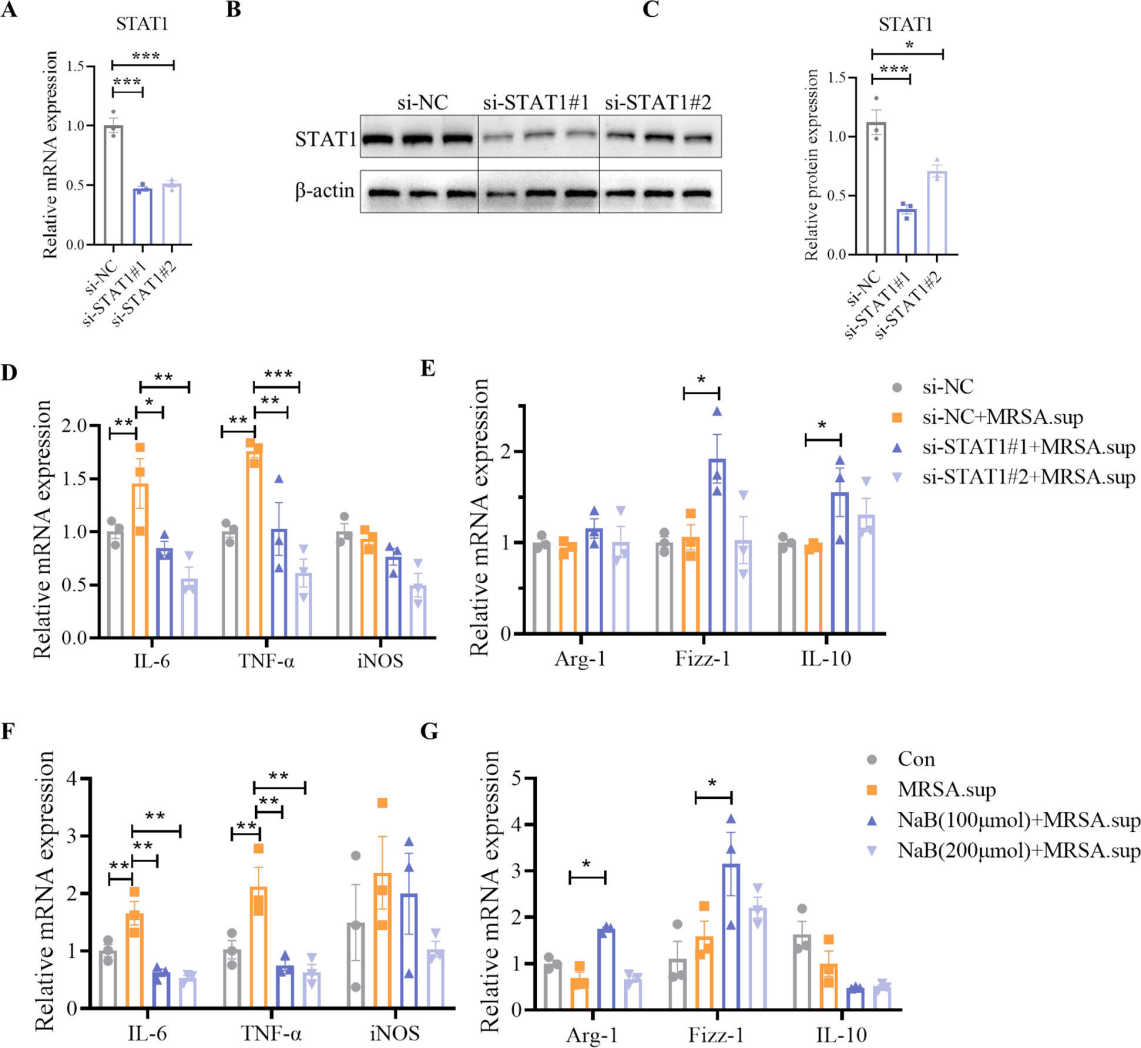
Sodium butyrate skews macrophage polarization toward M2 phenotype by suppressing STAT1. (**A–C**) Gene knockdown effect of STAT1 was verified by RT-PCR and western-blot. (**D–E**) The mRNA levels of M1 and M2 macrophage markers in MRSA.sup stimulated cells transfected with control siRNA (si-NC) or STAT1 siRNA. (**F–G**) The mRNA levels of M1 and M2 macrophage markers in MRSA.sup stimulated cells with or without NaB pretreatment. *, *P* < 0.05, **, *P* < 0.01, and ***,: *P* < 0.001 were calculated by one-way ANOVA with Duncan’s post hoc test.

**Fig 7:**
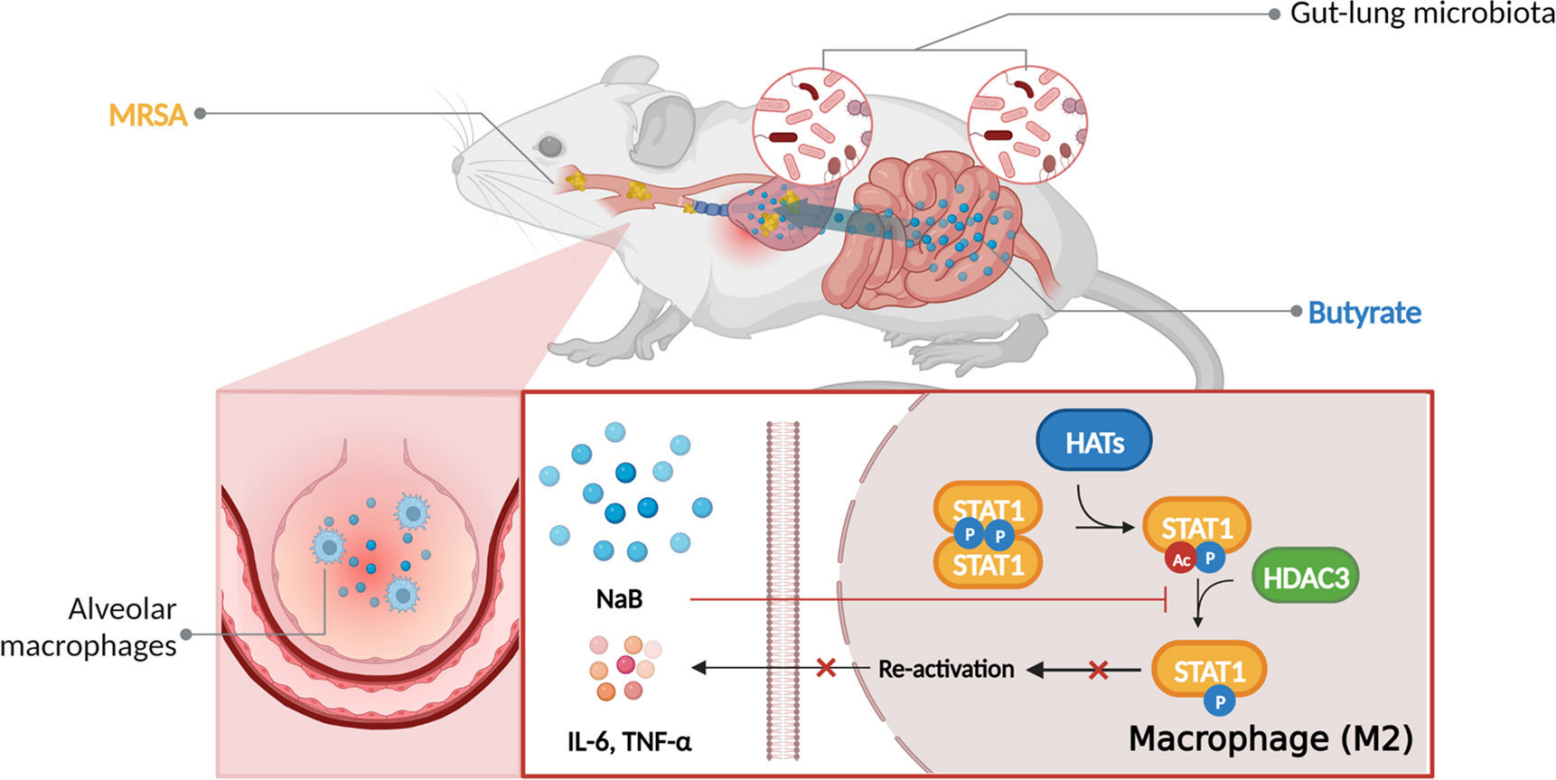
Schematic model of the protective effect of butyrate on MRSA pneumonia. MRSA pneumonia modifies the levels of lung and gut microbiota homeostasis, whereas butyrate prevents MRSA pneumonia through improving gut-lung microbiota. Mechanically, as an inhibitor of HDAC3, elevated circulation butyrate inhibits dimer STAT1 phosphorylation by promoting STAT1 acetylation, which then alters AM polarization to M2 phenotype to reduce lung inflammation.

Previous studies have indicated that STAT1 promotes the M1 polarization of macrophages (32). The knockdown efficiency of two siRNA sequences was assessed through western blot and qPCR, revealing that si-STAT1 #2 exhibited comparatively inferior efficiency compared to si-STAT1 #1 (Fig. 6A through C). siRNA for STAT1 significantly reduced the level of M1 macrophage markers (IL-6 and TNF-α) induced by MRSA, and increased the level of M2 macrophage markers (Fizz-1 and IL-10) (Fig. 6D and E). Pretreatment with NaB enhanced the expression of M2 macrophage markers (Arg-1 and Fizz-1) while decreasing the expression of M1 macrophage markers (IL-6 and TNF-α) at the transcriptional level (Fig. 6F and G). These findings indicate that NaB mitigated the STAT1-mediated inflammatory response and induced a shift in alveolar macrophage polarization toward the M2 phenotype (Fig. 7).

## DISCUSSION

Pneumonia induced by MRSA is a significant contributor to morbidity and mortality in hospitalized patients and tends to indicate a poor prognosis (33, 34). The discovery that gut microbiota can regulate the immune function of the lung through the gut-lung axis provides a novel strategy for managing lung infectious diseases (35). As summarized in Fig. 7, we here demonstrate that MRSA pneumonia leads to dysbiosis in the gut-lung microbiota and a reduction in systemic butyrate levels. And pretreatment of NaB is beneficial to ameliorate lung pathology through reshaping lung and gut microbiome. Additionally, NaB pretreatment elevates circulating butyrate levels and induces a shift in alveolar macrophage polarization toward the M2 phenotype by enhancing HDAC3-mediated STAT1 acetylation and subsequently suppressing STAT1 activation.

In the past few years, the dysbiosis of lung microbiota has been recognized as a consequence of various lung diseases (36, 37). Exposure to airborne fine particulate matter (PM2.5) has been shown to significantly alter the richness, evenness, and composition of the lung microbiome (38). Furthermore, investigations into the lung microbiota profiles of 20 deceased patients with coronavirus disease 2019 have revealed an imbalance characterized by the overabundance of *Acinetobacter* (39). In our study, the lung microbiota analysis unveiled noteworthy reductions in α-diversity and several beneficial bacterial genera following exposure to MRSA. These alterations in the lung microbiota could potentially contribute to the development of lung inflammation during MRSA infection. This observation suggests that the perturbation of the microbiota during MRSA infection leads to a decline in butyric acid content, which, in turn, may be implicated in lung inflammation.

It is widely accepted that many respiratory infections coincide with an imbalance in the intestinal microbiota. For instance, *Pseudomonas aeruginosa* infection pneumonia weakened the anti-infection or anti-inflammatory ability of gut symbiotic bacteria in mice (40). *Streptococcus* pneumoniae infection has been shown to cause a significant increase in the relative abundance of *Bacteroidetes* and a decrease in *Firmicutes* at the phylum level in the intestinal microbiota (41). Consistent with previous studies, our investigation revealed that during MRSA-induced pneumonia, the abundance of *Firmicutes* considerably decreased in the gut. Furthermore, in accordance with the lung microbiota findings, there is a noteworthy depletion of unclassified_f__*Lachnospiraceae,* a pivotal contributor to gut butyrate production. This decline in abundance is accompanied by reductions in both gut and serum butyrate levels, which exhibit a significant negative correlation with pulmonary inflammatory factors.

Butyric acid is an SCFA and one of the main metabolites of intestinal microbial fermentation of dietary fiber (42). Extensive research has highlighted the involvement of butyric acid in various pathological processes, including autoimmune, cancer, and neurological disease (43 – 45). Notably, NaB has been shown to significantly mitigate inflammation and dysfunction of the intestinal epithelial barrier in sodium trinitrobenzene sulfonate-induced models (46). The regulation of butyrate on distal immunity has been recently reported. In our experiment, NaB pretreatment decreased invasive bacteria in lung, which is supported by the findings of studies about the inhibitory effect of NaB on *S.aureus in vitro* (47, 48). Moreover, NaB effectively alleviated MRSA infection-induced lung inflammation and significantly reduced levels of inflammatory cytokines in the lung. These results are consistent with prior investigations wherein NaB administration via gavage suppressed inflammation in models of gram-negative LPS-induced ALI (15).

Supplementation of NaB in various disease models has demonstrated the potential to improve gut microbiota (49, 50). The prediction results of KEGG function of lung microbiota demonstrated that NaB significantly reversed the decrease in bacterial abundance related to cell migration caused by MRSA attack. For gut microbiota, NaB pretreatment reduced the increase of *Erysipelotrichaceae*_unclassified, which increased significantly in the gut microbiota of multiple sclerosis patients (51). Functional prediction showed that NaB pretreatment significantly decreased the abundance of potential pathogenic bacteria in the intestine. These findings suggest that the regulation of NaB on lung and gut microbiota may be helpful in alleviating lung injury.

Remarkably, NaB pretreatment resulted in increased levels of butyric acid in the gut and circulation compared to the MRSA infection group, suggesting that butyric acid may directly interact with lung cells. AMs serve as the initial line of defense against respiratory pathogen infection (52). The phenotypic transition of macrophage from anti-inflammatory (M2)-like to pro-inflammatory (M1)-like plays a crucial role in the macrophage function and its mediation of inflammatory response (53, 54). STATs are the most important transcription factors and play a major role in the immune response to pathogens (55). CBP and HDAC3 dynamically regulate the phospho-acetyl STAT1 signaling (31). Our results of alveolar macrophages *in vitro* demonstrated that NaB promotes STAT1 acetylation while inhibiting its phosphorylation through the disruption of the HDAC3-STAT1 interaction, thereby skewing macrophage polarization toward the M2 phenotype.

While we have demonstrated the protective effects of butyrate in MRSA pneumonia, it is essential to acknowledge that the current research does possess certain limitations, which necessitate further investigation. Firstly, our current results do not offer a specific explanation for the alterations in gut microbiota induced by MRSA pneumonia. Secondly, although the reduction in butyrate-producing microorganisms in the gut-lung axis has been observed, the principal driver of the decline in host butyrate levels remains undetermined, whether attributed to the pulmonary microbiota or the gut microbiota. Thirdly, while we have explored the *in vitro* mechanisms of butyrate salts, with a specific focus on their impact on alveolar macrophages, future experiments should also encompass other immune cells in the lungs for a comprehensive understanding.

In summary, our findings indicate that the administration of NaB demonstrates effective mitigation of MRSA-induced lung inflammation by modulating both the gut and lung microbiota. Furthermore, elevated circulating butyrate levels are associated with macrophage polarization toward an M2 phenotype. This study not only provides a novel perspective on the pathogenesis of MRSA pneumonia, but also emphasizes NaB as a promising preventive strategy against pneumonia.

## MATERIALS AND METHODS

### Mice

Male BALB/c mice, 6–8 weeks old and weighing 20 g–22 g, were provided by the Chengdu Dashuo Laboratory Animal Co., Ltd (Chengdu, China). Mice were housed for at least 1 week in the Experimental Center with a 12-h light and 12-h dark cycle prior to the experiment.

### Experimental design and animal treatments

For MRSA-induced pneumonia experimentation, mice were randomly assigned to two groups: (i) control group (Con, *n* = 8); (ii) MRSA treatment group (MRSA, *n* = 14). Mice were anesthetized with a mixture of 2,2,2-tribromoethanol and 2-methyl-2-butanol via intraperitoneal injections. Subsequently, MRSA suspension (5 × 10^6^ CFU) was performed through orotracheal intubation using a 20 G intravenous cannula (46). For the NaB prevention experimentation, mice were assigned randomly into three groups: (i) control group (Con, *n* = 10); (ii) MRSA treatment group (MRSA, *n* = 14); and (iii) NaB pretreatment group (NaB + MRSA, *n* = 14). After NaB (100 mg/kg) treatment via oral gavage for 10 days, the mice were instilled into the trachea with either saline solution or MRSA suspension (5 × 10^6^ CFU). Mice in each group were euthanized separately for 24 h after MRSA challenges. Blood and tissue samples were stored at −80°C.

The *Staphylococcus aureus* Mu50 strain utilized in this research was a clinical MRSA strain. Bacteria were cultivated overnight in tryptic soy broth (TSB) medium (Solarbio Scitech, Beijing, China), and the CFU were adjusted by measuring their optical density at 600 nm. For the cell test, the supernatant of MRSA (MRSA.sup) medium is from 10^8^ CFU bacterial supernatant. NaB was acquired from Sigma-Aldrich (St. Louis, MO, USA). Broad-spectrum antibiotics were acquired from Yuanye Biotech (Shanghai, China).

### Bronchoalveolar lavage

After the lung tissue was harvested, BALF was collected with an 8 g venous catheter by washing the lungs with 3 mL of ice-cold sterile phosphate-buffered saline (PBS). Fifty microliters of BALF was plated on TSB agar plates for colony counting. The remaining BALF was centrifuged (500 g for 10 min at 4°C), and stored at −80°C for 16S rDNA gene sequencing. According to the manufacturer’s recommendations, samples were sequenced on an Illumina NovaSeq platform which were provided by LC-Bio. All instruments were sterilized.

### HE staining

After lung lavage, the lower left lobes were fixed with 4% paraformaldehyde, embedded in paraffin, and sliced into 4 μm-thick sections. The lung tissue was then deparaffinized and stained using HE and light microscopy for further analysis (Servicebio, Wuhan, China). Remaining lung tissue was frozen in liquid nitrogen and stored at −80°C.

### Real-time quantitative PCR

Total RNA isolated from lung was extracted using Trizol Reagent (Invitrogen, Carlsbad, CA), and was then used for reverse transcription (RT) reaction. Complementary DNA synthesis was carried out using Moloney murine leukemia virus (M-MLV) reverse transcriptase following the manufacturer’s protocol (Thermo Fisher Scientific). Real-time PCR was performed using SYBR Premix Pro Taq HS qPCR AG11701 kit (Accurate Biotechnology, Changsha, China) in the iQ5 system (Bio-Rad, Hercules, CA). Cyclophilin (CYC) was used to quantify the target gene RNA, by comparative cycle threshold (CT) method (2^−ΔΔCt^). The sequences of the primers used for qPCR are listed in Table S1.

### DNA extractions and 16S rDNA sequencing

The BALF and cecum bacterial analysis was evaluated by 16S rRNA amplification of V3–V4 region and Illumina sequencing. Briefly, the total microbial genomic DNA of each sample was collected using E.Z.N.A. soil Kit (Omega Bio-tek, Norcross, GA, USA) following the manufacturer’s instructions. The concentration of DNA was assessed using Thermo NanoDrop2000 (Thermo Scientific, Wilmington, MA, USA), and quality of DNA was detected by 1.0% agarose gel electrophoresis. The V3–V4 regions of the 16S rRNA gene were amplified by PCR amplification (ABI 9700 GeneAmp), the primers were 338F 5´-ACTCCTACGGGAGGCAGCAG-3´ and 806R 5´-GGACTACHVGGGTWTCTAAT-3´. The PCR product was purified using AxyPrep DNA Gel Extraction Kit (Axygen Biosciences, Union City, CA, USA) and detected by 2% agarose. QuantiFluor-ST (Promega, USA) was used for detection and quantification. An equal amount of DNA was sequenced by Illumina MiSeq platform (Illumina, San Diego, USA) according to the standard protocols by Majorbio Bio-Pharm Technology Co. Ltd. (Shanghai, China). Negative controls for DNA extraction and PCR amplification, as well as a mock community, were included in each HiSeq run for quality control.

### SCFAs analysis

For the SCFAs measurements, the mouse cecum chyme and serum homogenized with ddH_2_O were centrifuged at 4°C, 13,500 g for 10 min. Supernatant was homogenized with 0.1 mL 25% metaphosphoric acid (Aladdin, Shanghai, China), incubated at 4°C for 4 h, and centrifuged at 4°C, 13,500 g for 15 min. An appropriate amount of supernatant was mixed with crotonic acid (10  g/L) and then filtered through a 0.45 µm microporous filtration membrane. The SCFAs were separated and quantified with an Agilent 7820 A GC system equipped with a polar capillary column (LanZhou Atech Technologies-free fatty acid phase (AE-FFAP), 30 m × 0.25 mm × 0.33 µm) and a flame ionization detector.

### Immunohistochemistry (IHC) analysis

Freshly isolated lung tissues from mice were fixed in 4% paraformaldehyde and air extraction for 24 h. Paraffin-embedded sections of lung were prepared for IHC staining. Citrate buffer solution (0.01 mol/L, PH = 6.0) was used for antigen retrieval under a high temperature and high pressure. The sections were incubated with 3% H_2_O_2_ solution, blocked with 5% bovine serum albumin (BSA) (Beyotime) for 30 min at room temperature, and incubated with a F4/80 antibody overnight at 4°C. After washing three times with PBS, sections were incubated with horseradish peroxidase (HRP)-labeled secondary antibody (ab270144; dilution, 1:100; Abcam) for 1 h at room temperature. For diaminobenzidine (DAB) color development, sections were stained with hematoxylin at room temperature for 3 min and observed under a light microscope after sealing.

### Western blot

Proteins in lung tissues and cells were lysed in radioimmunoprecipitation assay (RIPA) buffer (Beyotime Biotechnology, Shanghai, China) containing 1% phenylmethanesulfonyl fluoride, and protein concentration was quantified by BCA kit (Solarbio, Beijing, China). Subsequently, SDS-PAGE was used to separate the proteins, and then transferred to 0.22 µm polyvinylidene fluoride (PVDF) membranes (Roche, Basel, Switzerland). After blocking with 5% non-fat milk, membrane was incubated with the primary antibody overnight at 4°C. Thereafter, membrane was washed and incubated with a secondary antibody for 2 h at room temperature. Then the membrane was visualized using ECL Plus (Thermo Fisher Scientific, Waltham, MA) and the image was scanned using luminescent image analyzer (Bio-Rad, CA, USA). Antibodies used for western blot are listed in Table S2.

### Cell culture

The mouse alveolar macrophage line MH-S was purchased from the American Type Culture Collection and grown in Roswell Park Memorial Institute 1640 medium (Hyclone, Logan City, UT, USA) supplemented with 10% newborn calf serum (Hyclone). Cells were expanded in standard cell culture conditions: humidified atmosphere, 37°C, 5% CO_2_.

### ELISA

The inflammatory cytokines of lung were determined by ELISA kits (mlbio, Shanghai, China) according to the related instructions.

### Co-immunoprecipitation

Cultured MH-S cells were washed with 1× PBS and lysed in 500 µL Co-IP buffer (50 mM Tris, 150 mM NaCl, 0.5% Triton X-100, pH 7.5, protease inhibitor mix) for 30 min on ice. Cell lysate was then pre-cleared by centrifugation at 4°C, 12,000 g for 10 min. One-fifth of the total cell lysate sample was used for western blot analysis. The rest of the cell lysate was incubated with rabbit anti-STAT1 (3 µg) and IgG (2 µg) overnight at 4°C. The 10% protein-A/G agarose was washed three times with Co-IP buffer, 25 µL protein-A/G added to cell lysate, and incubated at 4°C for 2 h. The pellets were washed with Co-IP buffer three times and suspended in the SDS sample loading buffer. Finally, western blot analysis was performed.

### Statistical analysis

The sequencing analysis was conducted using the Illumina NovaSeq platform provided by Majorbio Bio-Pharm Technology Co. Ltd. (Shanghai, China). All of the data generated in the present study are expressed as mean ± standard error of the mean. Statistical analyses were performed by the Wilcox test, one-way ANOVA, or *t*-test using GraphPad Prism software (La Jolla, CA, USA). Statistically significant differences compared to controls are indicated as *P < 0.05, **P < 0.01, and ***P < 0.001.

## Data Availability

All data generated or analyzed during the current study are included in this published article (and supplemental files) or available in the NCBI Sequence Read Archive under accession numbers PRJNA762201, PRJNA918811, and PRJNA918852.

## SUPPLEMENTAL MATERIAL

Supplemental material mbio.01987-23-s0001.docx Fig. S1 to S5; Tables S1 to S2.

ASM does not own the copyrights to Supplemental Material that may be linked to, or accessed through, an article. The authors have granted ASM a non-exclusive, world-wide license to publish the Supplemental Material files. Please contact the corresponding author directly for reuse.
