# Supplementary material for: Butyrate protects against MRSA pneumonia via regulating gut-lung microbiota and alveolar macrophage M2 polarization
Source: mBio. 2023 Sep 27;14(5):e01987-23. doi: 10.1128/mbio.01987-23 (PMC10653920; doi:10.1128/mbio.01987-23)
Supplement: Supplemental material — Fig. S1 to S5; Tables S1 to S2. [file mbio.01987-23-s0001.docx]

**
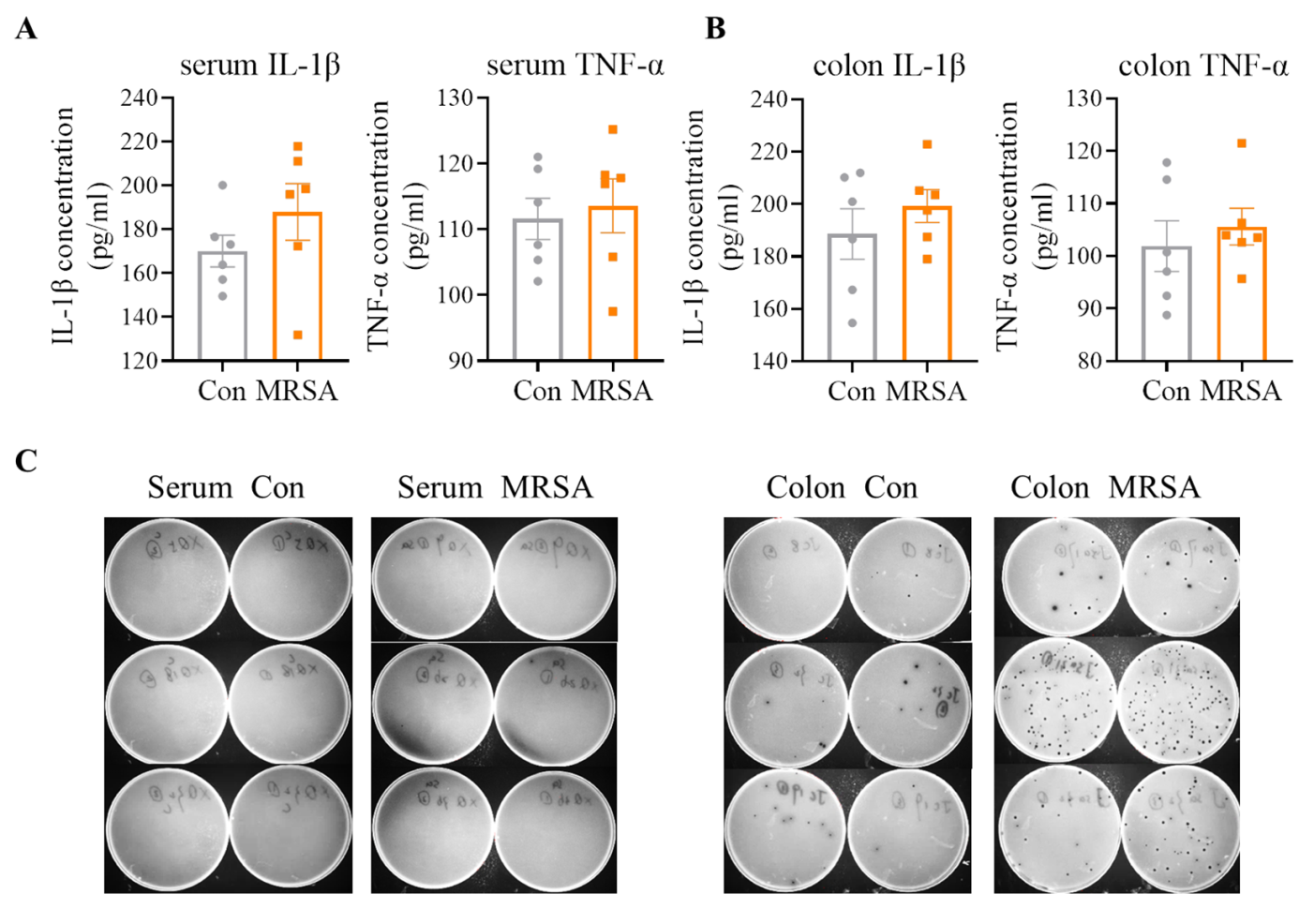
**

**Fig. S1** (A-B) ELISA results for IL-1β and TNF-α cytokine expression in serum and colon (n=6). (C) Plate count of *S. aureus* in serum and colon using selective culture medium.

**
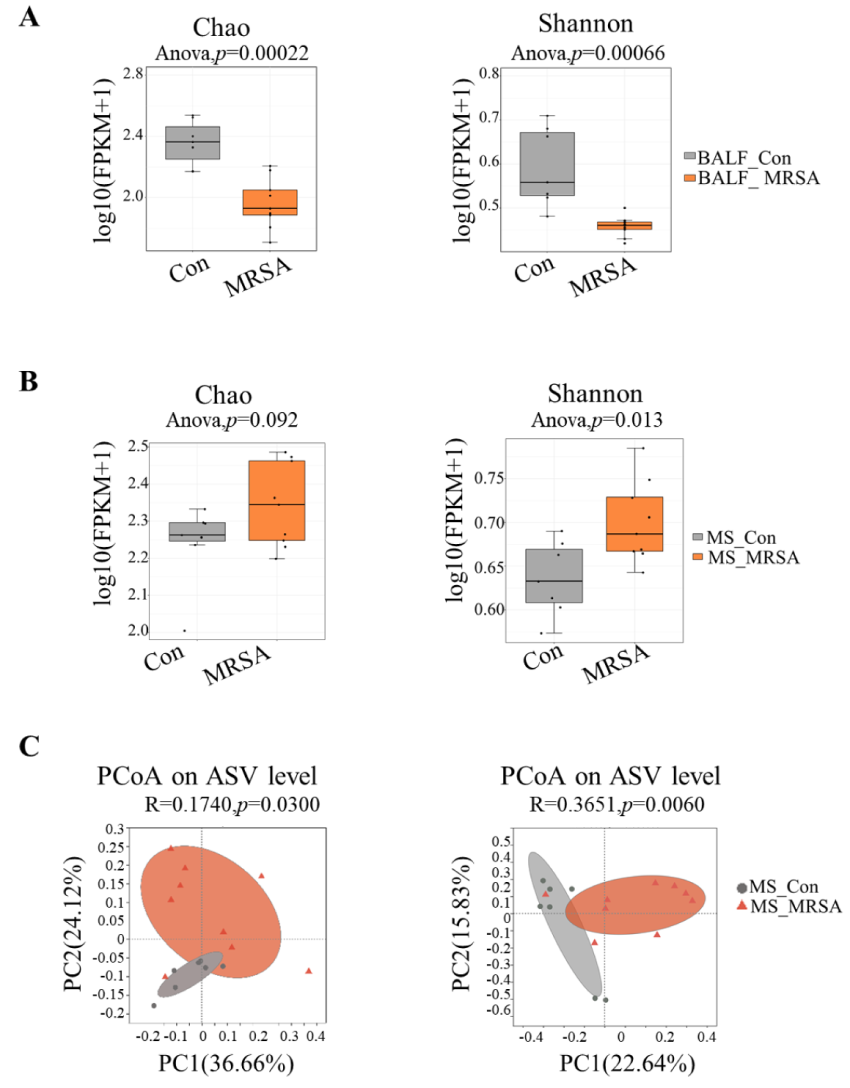
**

**Fig. S2** (A) Differences in α-diversity of lung microbiota. (B) Differences in α-diversity of gut microbiota. (C) Gut microbial β-diversity was calculated based on weighted (left) and bray-curtis (right) UniFrac metrics and presented as the principal coordinate analysis (PCoA) plot.

**
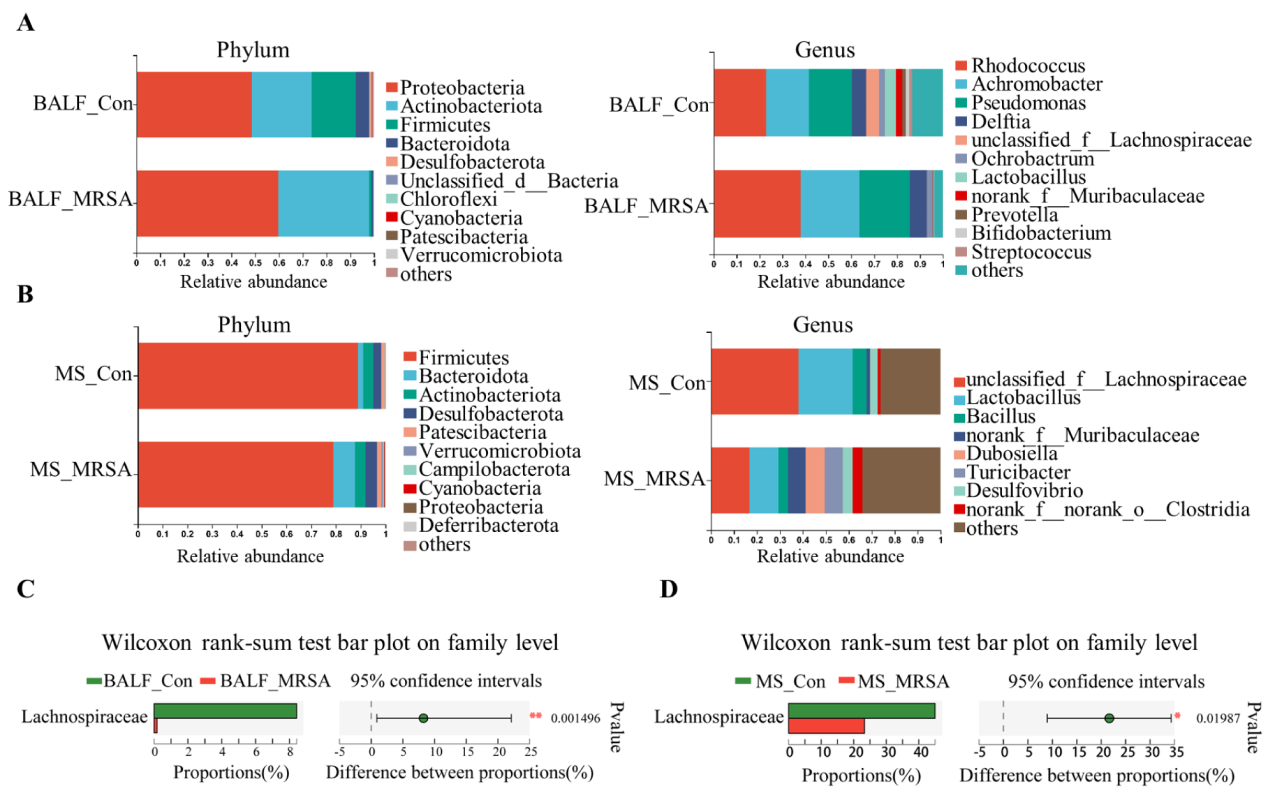
**

**Fig****. S3** (A-B) Lung and gut microbiota composition at the phylum and genus level.

**
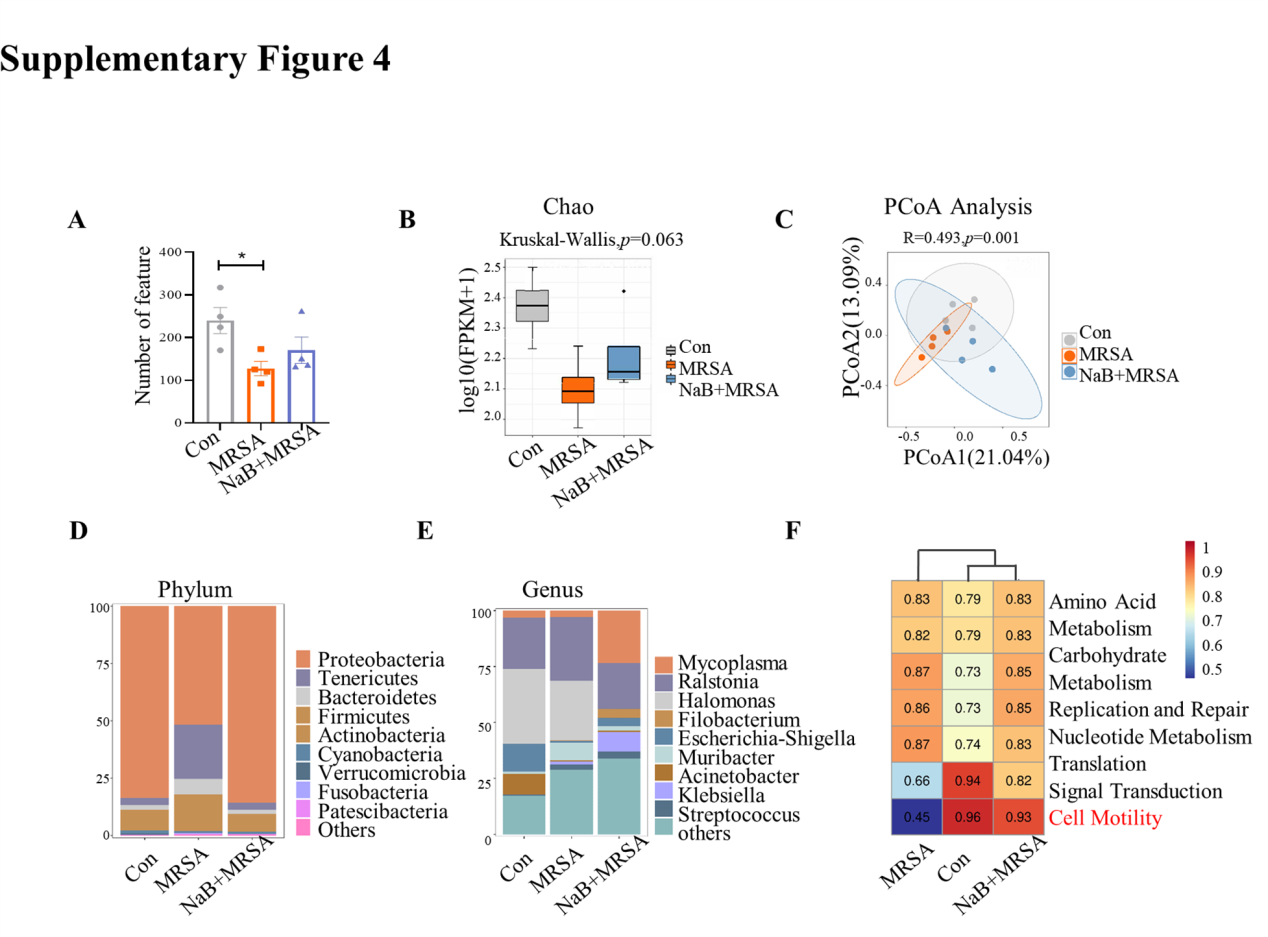
**

**Fig. S4 Effects of MRSA exposure and NaB pretreatment on lung microbiota.** (A) ASV numbers of lung microbiota in three groups. (B) Chao1 richness index. (C) PCoA score plot of lung microbiota in mice of three groups. (D-E) Lung microbiota composition at the phylum and genus level. (F) Abundance of lung bacterial function groups at level 3 predicted with the Tax4Fun tool relative to treatment. *: *P* < 0.05, was calculated by one-way ANOVA with Duncan’s post hoc test.

**
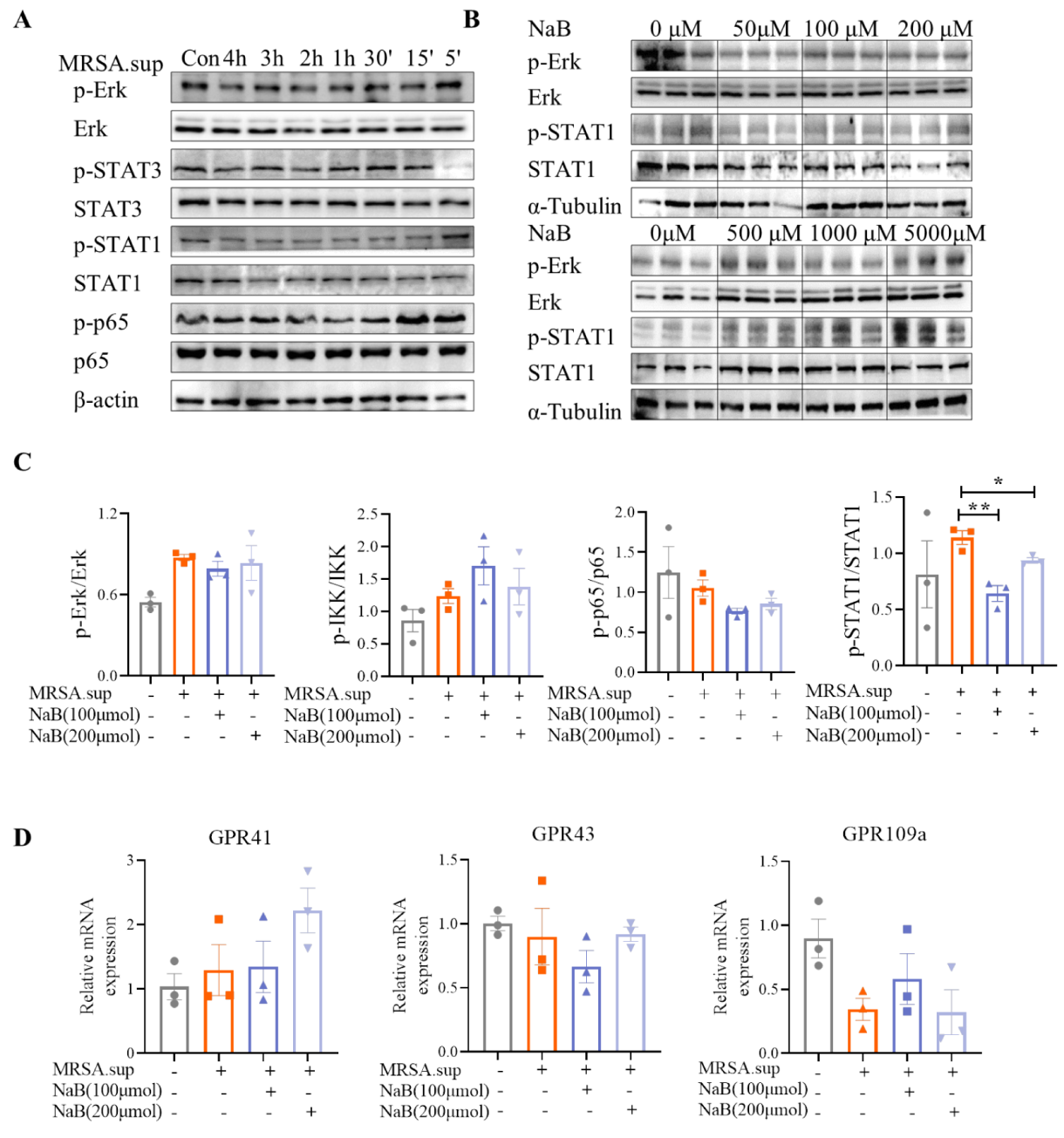
**

**Fig. S5** (A) Representative western blot bands of time course of inflammation related proteins in heat-inactived MRSA induced MH-S. (B) Representative western blot bands of the p-Erk, Erk, p-STAT1, and STAT1 after treatment of MH-S with different concentrations of NaB. (C) Quantitative analyses of inflammation related proteins in MRSA.sup and NaB+ MRSA.sup induced MH-S. (D) The mRNA levels of GPCRs were detected by RT-qPCR. *: *P* < 0.05, **: *P* < 0.01, were calculated by one-way ANOVA with Duncan’s post hoc test.

**Table S1**

Sequences of qPCR primers

| Gene | Forward 5'-3' | Reverse 5'-3' |
| --- | --- | --- |
| TNF-α | CCTGTAGCCCACGTCGTAG | GGGAGTAGACAAGGTACAACCC |
| IL-1β | GAAATGCCACCTTTTGACAGTG | TGGATGCTCTCATCAGGACAG |
| IL-6 | CAAAGCCAGAGTCCTTCAGAG | GCCACTCCTTCTGTGACTCC |
| cyc | GCATACAGGTCCTGGCATC | CACCTTCCCAAAGACCACA |
| Arg-1 | GGAATCTGC ATGGGCAACCTGTGT | AGGGT CTACGTCTCGCAAGCC |
| Fizz-1 | CCAATCCAGCTAACTATCCCTCC | ACCCAGTAGCAGTCATCCCA |
| IL-10 | CTTACTGACTGGCATGAGGATCA | GCAGCTCTAGGAGCATGTGG |
| iNOS | TCCTCACTGGGACAGCACAGA | GTGTCATGCAAAATCTCTCC |

**Table** **S2**

List of antibodies used for immunohistochemistry or western blot

| Antibody | Supplier | Species | Dilution |
| --- | --- | --- | --- |
| F4/80  p-STAT3  STAT3  p-STAT1  STAT1  p-ERK  ERK  p-p65  p65  Arg-1  β-actin | Affinity  Cell Signaling  Proteintech  Cell Signaling  Proteintech  Proteintech  Proteintech  Proteintech  Proteintech  PTM BIO  ABclonal | Rabbit  Rabbit  Rabbit  Rabbit  Rabbit  Rabbit  Rabbit  Rabbit  Rabbit  Rabbit  Mouse | 1:150  1:1000  1:1000  1:1000  1:1000  1:1000  1:1000  1:1000  1:1000  1:500  1:10000 |
